# Is Body Fat a Predictor of Race Time in Female Long-Distance Inline Skaters?

**DOI:** 10.5812/asjsm.34853

**Published:** 2010-09

**Authors:** Beat Knechtle, Patrizia Knechtle, Thomas Rosemann, Romuald Lepers

**Affiliations:** 1 Gesundheitszentrum St. Gallen, St. Gallen, Switzerland; 2 Institute of General Practice and for Health Services Research, University of Zurich, Zurich, Switzerland; 3 INSERM U887, Faculty of Sport Sciences, University of Burgundy, Dijon, France

**Keywords:** Skinfold thickness, Physical Endurance, Body Fat, Skating, Training volume

## Abstract

**Purpose:**

The aim of this study was to evaluate predictor variables of race time in female ultra-endurance inliners in the longest inline race in Europe.

**Methods:**

We investigated the association between anthropometric and training characteristics and race time for 16 female ultra-endurance inline skaters, at the longest inline marathon in Europe, the ‘Inline One-eleven’ over 111 km in Switzerland, using bi- and multivariate analysis.

**Results:**

The mean (SD) race time was 289.7 (54.6) min. The bivariate analysis showed that body height (r=0.61), length of leg (r=0.61), number of weekly inline skating training sessions (r=-0.51) and duration of each training unit (r=0.61) were significantly correlated with race time. Stepwise multiple regressions revealed that body height, duration of each training unit, and age were the best variables to predict race time.

**Conclusion:**

Race time in ultra-endurance inline races such as the ‘Inline One-eleven’ over 111 km might be predicted by the following equation (r^2^=0.65): Race time (min)=-691.62+521.71 (body height, m)+0.58 (duration of each training unit, min)+1.78 (age, yrs) for female ultra-endurance inline skaters.

## INTRODUCTION

In endurance athletes, the association between anthropometric characteristics such as body mass, body height, body mass index, length and circumferences of extremities, body fat and skin-fold thicknesses and performance have been investigated mainly in disciplines of swimming, cycling and running. Body mass was related to performance in runners^[1–3]^, and both road^[4]^ and off-road cyclists ^[5, 6]^. Indeed, athletes with a lower body mass had an advantage in climbing. In addition, body height was shown to be associated with swimming performance^[7–9]^. Body mass index^[10]^ and circumference of upper arm^[3, 11]^ were also shown to be related to performance in ultra-endurance runners. Besides, body fat showed an association with performance in female marathon runners^[12]^, in male ultra-marathoners^[13]^ and in female swimmers^[8, 14]^. On the other hand, the sum of five skin-fold thicknesses was related to performance in male 10 km runners^[15]^, and the sum of seven skin-folds was correlated to performance time in male marathoners^[1]^. Regarding selected skin-fold thicknesses, front thigh and medial calf skin-fold were related to 10 km running times in elite male runners, and iliac crest and abdominal skin-fold thicknesses were associated with marathon race times in female elite runners^[16]^.

Volume and intensity of training influence performance in endurance athletes as well, apart from anthropometric characteristics. In marathoners, the longest mileage covered per training session was the best predictor of successful completion of a marathon^[17]^. Runners being trained for more than 100 km per week had significantly faster race times over 10 km to 90 km than athletes covering less than 100 km^[18]^. Elite male runners with a higher training frequency, a higher weekly training volume and a longer running experience had a better 10 km performance^[15]^. Hewson and Hopkins showed a correlation between seasonal weekly duration of moderate continuous running and performance of runners specialising in longer distances^[19]^. Top class marathon runners were trained for more total kilometres per week, and at a higher velocity, than runners at a lower level^[20]^. Peak running velocity in training was highly related to 5 km run times for both male and female athletes^[21]^.

The association between anthropometric and training characteristics and performance has been investigated in disciplines of above mentioned sports such as swimming, cycling, running and triathlon for both male and female athletes, but not for female long-distance inline speed skaters. The aim of this study was to investigate the association between anthropometric and training characteristics and race time in female athletes at the longest inline marathon in Europe, the ‘Inline One-eleven’, in Switzerland. We hypothesised that body fat would be related to race time in female long-distance inline skaters.

## METHODS AND SUBJECTS

**Race:** The organiser of the ‘Inline One-eleven’ in St. Gallen, Switzerland, contacted all the participants of the race via a separate newsletter upon inscription to the race in 2009, the 12^th^ year of this event. The ‘Inline One-eleven’ was the longest inline skating race in Europe, covering a total distance of 111 km, with a total altitude of 1,400 m to climb. The start of the race was in the heart of the City of St. Gallen, and then went on a large loop of 111 km in the East of Switzerland returning to St. Gallen. Inliners from all over Europe came to St. Gallen for the longest inline race in Europe, held on completely closed routes.

**Subjects:** A total of 16 female athletes volunteered for this investigation. They all gave their written informed consent. The study was approved by the Institutional Review Board for Use of Human Subjects of St. Gallen, Switzerland. The athletes came on 15 August 2009 to get their race numbers and instructions for the race. On 16 August 2009 at 07:00 a.m. they started the race. During the 111 km, the organiser offered 11 refreshment points, including the opportunity to repair the inline shoes in case of a malfunction. Total race time was measured using an electronic chip system.

**Measurements and Calculations:** The day before start of the race body mass, body height, length of limbs, circumference of limbs and hip, and the thickness of skin-folds (pectoralis, axillar, triceps, subscapular, abdomen, suprailiac, thigh and calf) were measured. With this data, body mass index, the sum of eight skin-folds and percent body fat were calculated. Body mass was measured using a commercial scale (Beurer BF 15, Beurer, Ulm Germany) to the nearest 0.1 kg. Body height was measured using a stadiometer to the nearest 0.5 cm. Skin-fold data was obtained using a skin-fold calliper (GPM-Hautfaltenmessgerät, Siber & Hegner, Zurich, Switzerland) and recorded to the nearest 0.2 mm. One trained investigator took all the measurements as inter-tester variability is a major source of error in skin-fold measurements. Skin-fold thicknesses were determined on the right side of the body in all athletes. The skin-fold measurements were taken three times and the mean was then used for the analyses. The skin-fold measurements were standardised to ensure reliability and readings were performed 4 s after applying the calliper, according to Becque et al^[22]^. An intra-tester reliability check was conducted on 27 male runners prior to testing. No significant difference was observed between the two trials according to the sum of skin-folds (*P*>0.05). The intra-class correlation (ICC) was high at *r*=0.95. The same investigator was also compared with another trained investigator to determine objectivity. No significant difference existed between the testers (*r*=0.97; *P>*0.05) (data is under revision for submission). Percentage of women's body fat was calculated using the following anthropometric formula according to Ball et al. ^[23]^:

$$\text{Body fat}(\text{percent})=-6.40665+0.41946(\sum 3\text{SF})-0.00126{\left(\sum 3\text{SF}\right)}^{2}+0.12515(\text{hip})+0.06473(\text{age})$$

**Training diary:** Upon inscription to the study, until the start of the race, the athletes were asked to maintain a comprehensive training diary recording training sessions showing distance and duration in the preparation for the race. The training record consisted of the number of weekly training units of inline skating, showing duration, kilometres and pace. Furthermore, they reported the number of years that they had been active inliners, including participation in inline races.

**Statistical Analysis:** Data is presented as mean and standard deviation (SD). Bivariate correlation analysis between the anthropometric characteristics such as body mass, body height, body mass index, circumference and length of limbs, body fat, sum of skin-folds, selected skin-fold thicknesses at abdomen, iliac crest, front thigh and medial calf, and training characteristics such as its volume and intensity with race time were performed using Pearson correlation analysis. These variables had been investigated, in the available literature, in male and female endurance athletes. Stepwise multiple regression analysis was then used to determine the best variables correlated with the race time. An alpha level of 0.05 was used to indicate significance.

## RESULTS

All 16 female participants completed the ‘Inline one-eleven’ and they finished within 289.7 (54.6) min. In the bivariate analysis (Table 1), the anthropometric characteristics of body height and length of leg were related to total race time. For the training characteristics, the number of weekly training units and the duration of each training session of inline skating were related to race time (Table 2). Stepwise multiple regressions showed that body height, duration of each training unit, and age were the best variables correlated with race time. Race time of ‘Inline one-eleven’ might be predicted by the following equation (r^2^=0.65) for female athletes: Race time (min)=-691.62+521.71 (body height, m)+0.58 (duration per training unit, min)+1.78 (age, yrs).

**Table 1:** Age, anthropometric and training characteristics of the subjects and their correlation with race time

Age and anthropometric characteristics	Mean (SD)	r*
**Age (y)**	36.7 (10.0)	0.44
**Body height (m)**	1.66 (0.04)	0.61, *P* =0.011
**Body mass (kg)**	60.6 (6.4)	0.25
**Body mass index (kg/m**^**2**^**)**	21.9 (2.0)	−0.09
**Length of leg (cm)**	82.2 (2.7)	0.61, *P* =0.012
**Length of arm (cm)**	74.4 (2.8)	0.33
**Circumference upper arm (cm)**	26.4 (1.9)	−0.01
**Circumference thigh (cm)**	55.7 (3.3)	−0.25
**Circumference calf (cm)**	36.6 (2.3)	0.20
**Skin-fold iliac crest (mm)**	17.4 (6.9)	−0.03
**Skin-fold abdomen (mm)**	14.1 (5.2)	−0.01
**Skin-fold front thigh (mm)**	27.3 (6.5)	−0.08
**Skin-fold medial calf (mm)**	10.3 (3.4)	0.05
**Sum of eight skin-folds (mm)**	103.1 (23.9)	−0.11
**Body fat (%)**	27.8 (3.8)	−0.04

* P-value is given in case of a significant correlation

**Table 2:** Training characteristics of the subjects and their correlation with race time

Training characteristics	Mean (SD)	r*
**Years of being an active inliner**	8.1 (5.4)	−0.48
**Number of weekly inline skating training sessions**	2.5 (1.0)	−0.51, *P=*0.045
**Distance of each inline skating training session (km)**	31.2 (7.3)	0.42
**Duration of each inline skating training session (min)**	85.2 (36.4)	0.61, *P=*0.013
**Speed of inline skating during each training session (km/h)**	22.4 (4.6)	−0.32

* P-value is given in case of a significant correlation

## DISCUSSION

The aim of this study was to investigate the association between anthropometric and training characteristics and female athletes’ race time in an ultra-endurance inline skating race, such as the ‘Inline one-eleven’. We hypothesised that body fat would be related to race time in female long-distance inline skaters.

**Anthropometry:** In contrast to our hypothesis, body fat was not related to race time. The relation between the physical characteristics such as body mass^[1–6, 24]^, body fat^[3, 13, 25]^ and skin-fold thicknesses^[15, 16, 26–28]^ and endurance performance has mainly been investigated in runners, cyclists and triathletes. Body fat showed a significant positive correlation with male ultra-marathoners performance time in a 161-km trail ultra-marathon^[13]^. However, this was not the case for 100 km ultra-marathoners^[29]^ or male 24-hour ultra-marathoners^[30]^. Body fat was not related to race time in female ultra-endurance inliners in this study either.

Apart from body fat, the association between skin-fold thicknesses and endurance performance has been investigated in runners over distances from 100 m to ultra-endurance. In male 10 km runners, the total sum of five skin-fold thicknesses was related to performance^[15]^. Additionally, in male marathoners, the sum of seven skin-folds was correlated to marathon performance times^[1]^. For female runners, the iliac crest skin-fold (r=0.62) and the abdominal skin-fold (r=0.61) were related to marathon performance times in highly trained runners ^[16]^. However, the length of a running performance may determine whether skin-fold thicknesses are related to performance or not. In male ultra-endurance runners skin-fold thicknesses showed no association with performance during a 24 hour run ^[30]^. Besides, the sum of skin-folds was not associated with race performance during a 100 km run ^[29]^. Neither the sum of skin-folds nor selected skin-folds of the lower limbs were related to the race time in female ultra-endurance inliners of the present study.

Type of performance also seems to influence the association between skin-fold thicknesses and performance. For instance no association between skin-fold thicknesses and race performance has been found in cyclists, neither in ultra-endurance road cyclists^[26]^, nor in ultra-endurance mountain-bikers^[28]^. However, in ultra-endurance triathletes competing in distances longer than the Ironman distance, the sum of eight skin-fold thicknesses was related to race performance^[27]^.

**Training:** In the bivariate analysis, the number of weekly training units and the duration of each training unit of inline skating were related to race time. The stepwise multiple regression showed that duration of each training unit of inline skating was the only training variable significantly correlated to race time apart from age and body height. We must assume that duration of training was of higher importance than training speed. Different studies of runners described that volume of training was important for performance as well^[17, 19, 20, 31]^. McKelvie et al. described that training pace was important for runners and speedier workouts were associated with faster marathon times^[31]^. Billat et al. concluded that top class marathon runners were trained for more total kilometres, and at a higher velocity, compared with high-level marathon runners^[20]^. Hewson and Hopkings showed a correlation between seasonal mean weekly duration of moderate continuous running, for runners specialising in longer distances, and performance in both male and female distance runners^[19]^. For marathoners, the longest mileage covered per training session was the best predictor of a successful completion of a marathon^[17]^.

**Limitations of the study:** This investigation is limited to the rather small sample size of 16 female ultra-endurance athletes. However, other studies on female ultra-endurance athletes were not able to recruit more female athletes, even at events with more participants. For instance, in an Ironman triathlon with more than 2,000 starters, only 16 female triathletes volunteered to participate in a study^[32]^. Furthermore, in a 100 km ultra-marathon with more than 1,500 starters, only 11 female runners participated in a study^[33]^. Therefore, the number of 16 female subjects in a study on ultra-endurance athletes seems quite representative due to the low number of female athletes generally participating in ultra-endurance races^[34]^. A further limitation is the fact that food and fluids intake was not recorded. In actual concept of exercise-associated hyponatremia, female gender is considered as a risk factor^[35]^. However, regarding female ultra-endurance runners, no female ultra-runner developed exercise-associated hyponatremia in a 100 km ultra-marathon^[33]^.

## CONCLUSION

To summarise, both anthropometric and training characteristics were related to race time in these female long-distance inline skaters. Race time in an ultra-endurance inline race such as the ‘Inline One-eleven’ might be predicted by the following equation (r^2^=0.65) for future participants: Race time (min)=-691.62+521.71 (body height, m)+0.58 (duration per training unit, min)+1.78 (age, yrs) for female ultra-endurance inline skaters.
